# Voltage-Dependent Modulation of Cardiac Ryanodine Receptors (RyR2) by Protamine

**DOI:** 10.1371/journal.pone.0008315

**Published:** 2009-12-15

**Authors:** Paula L. Diaz-Sylvester, Julio A. Copello

**Affiliations:** Department of Pharmacology, Southern Illinois University School of Medicine, Springfield, Illinois, United States of America; University of Virginia, United States of America

## Abstract

It has been reported that protamine (>10 µg/ml) blocks single skeletal RyR1 channels and inhibits RyR1-mediated Ca^2+^ release from sarcoplasmic reticulum microsomes. We extended these studies to cardiac RyR2 reconstituted into planar lipid bilayers. We found that protamine (0.02–20 µg/ml) added to the cytosolic surface of fully activated RyR2 affected channel activity in a voltage-dependent manner. At membrane voltage (V_m_; SR lumen - cytosol) = 0 mV, protamine induced conductance transitions to several intermediate states (substates) as well as full block of RyR2. At V_m_>10 mV, the substate with the highest level of conductance was predominant. Increasing V_m_ from 0 to +80 mV, decreased the number of transitions and residence of the channel in this substate. The drop in current amplitude (full opening to substate) had the same magnitude at 0 and +80 mV despite the ∼3-fold increase in amplitude of the full opening. This is more similar to rectification of channel conductance induced by other polycations than to the action of selective conductance modifiers (ryanoids, imperatoxin). A distinctive effect of protamine (which might be shared with polylysines and histones but not with non-peptidic polycations) is the activation of RyR2 in the presence of nanomolar cytosolic Ca^2+^ and millimolar Mg^2+^ levels. Our results suggest that RyRs would be subject to dual modulation (activation and block) by polycationic domains of neighboring proteins via electrostatic interactions. Understanding these interactions could be important as such anomalies may be associated with the increased RyR2-mediated Ca^2+^ leak observed in cardiac diseases.

## Introduction

In striated muscle, electrical excitation activates ryanodine receptors (RyR) located in the sarcoplasmic reticulum (SR) membrane which in turn mediate the massive release of intracellular Ca^2+^ required for activating the contractile system [1], [2], [3]. Electron microscopy studies indicate that cardiac (RyR2) channels could interact among themselves as they are physically connected in organized arrays at the terminal cisternae of SR [4]. Indeed, it has been shown that multiple RyRs synchronously activate and deactivate during excitation-contraction (EC)-coupling [1], [5], [6], [7], [8]. Moreover, under resting conditions, brief elementary events of Ca^2+^ release (“Ca^2+^ sparks”) arise as a result of the concerted activation and deactivation of six to twenty RyR2 in brief bursts lasting ∼5–20 ms [9], [10]. These functional channel-channel interactions seem to survive isolation and reconstitution in bilayers, where multiple RyRs often display synchronicity named “coupled gating” [11], [12], [13], [14]. It is also apparent that, in the cytosolic environment, RyR1 and RyR2 may be modulated via physical interactions with other associated proteins, such as the L-type Ca^2+^-channels [8], [15]. The nature of the interactions between neighboring RyRs and/or with associated proteins has not been fully defined, but it is likely that electrostatic interactions may play a role as the vestibule of RyRs contain negatively charged regions that could be a target for cationic ligands [16], [17]. Indeed, it is well known that RyR channel function can be modulated by positively charged moieties, including polycationic peptides such as protamine, histone and polylysine, which seem to display a variety of actions including activation and block of RyR-mediated Ca^2+^ release [18], [19], [20], [21]. Furthermore, in failing heart as well as in skeletal muscle pathologies, RyR-mediated SR Ca^2+^ release was found to have increased sensitivity to activation by polylysine [22], [23].

Protamine is a mix of highly cationic (arginine rich) peptides with molecular weight of ∼5.1 kDa (major component) which has been previously used as a tool to study how RyRs are modulated through electrostatic interactions [24], [25]. In this early study, large doses of protamine (>20 µg/ml) were found to fully inhibit skeletal (RyR1) channels regardless of the cytosolic Ca^2+^ levels [24]. We extended these studies to cardiac RyR2 reconstituted into planar lipid bilayers and tested a wider range of protamine levels (0.02 to 20 µg/ml). Our results indicate that the action of protamine added to the cytosolic surface of RyR2 is complex. It includes voltage-dependent activation and block as well as transitions to subconductance states (substates). Some of the results have been presented in preliminary form [26].

## Methods

### Drugs and Chemicals

CaCl_2_ standard for calibration was from Word Precision Instruments Inc. (Sarasota, FL). Phospholipids were obtained from Avanti (Alabaster, AL). Ryanodine was from Calbiochem (San Diego, CA). Imperatoxin A (IpTx_A_) was from Alomone Labs (Jerusalem, Israel). Ryanodol was obtained from hydrolyzed ryanodine as previously described [27]. Protamine and all other drugs and chemicals were either from Sigma-Aldrich or were reagent grade.

### Sarcoplasmic Reticulum Microsomes

All procedures with animals were designed to minimize pain and suffering and conformed to the guidelines of the National Institutes of Health. The committee on the Use and Care of Laboratory Animals of Southern Illinois University School of Medicine reviewed and approved the protocols for animal use. Sarcoplasmic reticulum (SR) microsomes were obtained from pig heart ventricle using heart homogenization and ultracentrifugation steps that follow the procedures published by Chamberlain *et al.* [28]. SR pellets obtained after high speed centrifugation were resuspended in 290 mM sucrose - 5 mM Imidazole buffer (pH = 7) and were aliquoted in cryovials (300 µl each) and kept in liquid nitrogen (better and safer long-term storage). Every month, a few cryovials are used to generate smaller aliquots of membranes (15 µl each) which were stored at –80°C for easy access. For experiments, aliquots were quickly thawed in water, kept on ice and used within 3–5 hours.

### Bilayer Technique

Reconstitution of RyR2 in planar lipid bilayers, was performed as previously described [29]. Briefly, planar lipid bilayers were formed on 80 to 100 µm-diameter circular holes in teflon septa, separating two 1.3 ml compartments. The *trans* compartment was filled with HEPES-Ca^2+^ solution containing HEPES 250 mM and Ca(OH)_2_ 53 mM, pH 7.4. The *trans* compartment was clamped at 0 mV using an Axopatch 200B patch-clamp amplifier (Axon Instruments, Foster City, CA). The *cis* compartment (ground) was filled with HEPES-Tris solution containing HEPES 250 mM and TrisOH 118 mM, pH 7.4. Bilayers of a 5∶4∶1 mixture of bovine brain phosphatidylethanolamine, phosphatidylserine and phosphatidylcholine (45–50 mg/ml in decane) were painted onto the holes of teflon septa from the *cis* side. Sarcoplasmic reticulum microsomes (5–15 µg) were then added to the *cis* solution followed by 500–1000 mM CsCl and 1 mM CaCl_2_ to promote vesicle fusion. After RyR currents (or Cl^−^ currents >100 pA at 0 mV) were observed, the *cis* chamber was perfused with HEPES-TRIS solution for 5 min at 4 ml/min. A mixture of BAPTA and dibromo-BAPTA was used to buffer free [Ca^2+^] on the cytosolic surface of the channel ([Ca^2+^]_cyt_) [29]. As previously done [29], RyR channels were identified by current amplitudes (∼3.5 pA at 0 mV), slope conductance (∼100 pS), reversal potential (∼−45 mV, *trans* - *cis*) and response to diagnostic ligands (e.g., ryanodine, Ca^2+^, ATP, caffeine and Ruthenium Red). RyR channel currents are depicted as positive (upward deflections of the current) in figures and reflect cation flux from the *trans* (lumenal) to the *cis* (cytosolic) compartment. Membrane voltages always represent the difference between *trans - cis* compartments (in mV).

### Single Channel Analysis

Channel currents were first filtered through the Axopatch 200B low-pass Bessel filter at 2 kHz, digitized at 20 kHz with an analog to digital converter (Digidata 1320, Axon Instruments) and stored on DVD. Recordings were analyzed using pClamp9 software (Axon Instruments). Analysis with this program included open times, closed times and open probabilities (P_o_), which were determined by half-amplitude threshold analysis of single-channel recordings as done before [29].

Recordings were digitally filtered at 500 Hz in order to estimate the probabilities of substates using two different methods:

#### 1) Manual analysis of the traces

Frame by frame analysis (100 ms/frame) was performed using pClamp9 on each 4-min recording. In most of our recordings, we were able to distinguish different levels of current that included the baseline, full openings and substates induced by protamine, ryanodol or imperatoxin A. Events, at different levels of current lasting more than 3 ms, were manually selected. The parameters (mean amplitude and duration) of each event were collected and averaged. The probability of substate occurrence, P_substate_, was estimated from the ratio: time spent in substate/total recording time. P_full open_ was estimated as the fraction of time spent in the maximal current level (full opening).

#### 2) Current-amplitude distributions

All-points current-amplitude histograms (band width = 0.01 pA) were obtained from each 4-min recording. Histograms were normalized so that total histogram area = 1. In most of our experiments, we were able to detect peaks (components) corresponding to the baseline, full opening and substate levels. Each component was fitted with a Gaussian function using the Levenberg-Marquardt method. When the fitting is good, the fitted area of each component can be used as an estimation of its probability.

In some cases, the small signal-to-noise ratio precluded the unequivocal resolution of all individual components from the amplitude distribution histograms. In these cases, the P_substate_ were only estimated using frame by frame analysis. It is important to point out that in previous studies [18] we have established that frame by frame analysis of higher signal-to-noise recordings yielded the same results as histograms (i.e. we can be confident when comparing P_o_ values calculated using different methods).

### Statistical Analysis

Data are shown as means±S.E.M. of *n* measurements. Statistical comparisons between groups were performed with Student's t-test of paired differences. Differences were considered statistically significant at P<0.05.

## Results

In this work, we studied the action of protamine added to the cytosolic surface of cardiac RyR2 reconstituted into planar lipid bilayers. High concentrations of protamine (>20 µg/ml) have been tested on partially active skeletal RyR1 channels [24]. Here, we extended these studies testing the effect of lower levels of protamine (0.02–20 µg/ml). As previous studies reported an inhibitory effect of protamine [19], [24], we conducted most of our experiments using fully activated RyR2 (locked open by the combined effect of high cytosolic Ca^2+^ and caffeine) in order to minimize the interference of RyR2 intrinsic gating events (closures) with the expected effect of protamine (full or partial block). However, other studies have suggested that polycationic peptides (histone, polylysine) could induce SR Ca^2+^ release under cellular resting conditions [19]. Consequently, we also tested the effect of protamine on RyR2 with low P_o_ at low (resting) cytosolic Ca^2+^ levels.

### Protamine Induces Block and Substates

**Figure 1:**
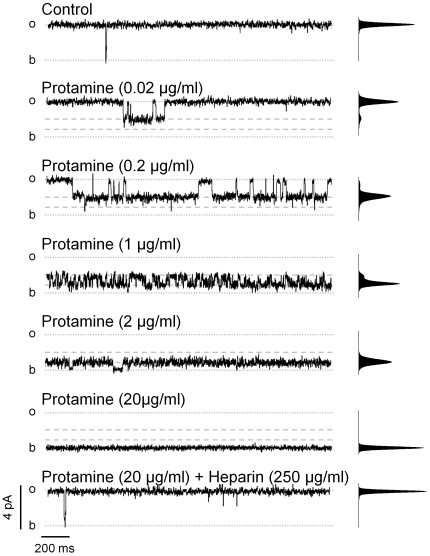
Effect of protamine on fully activated RyR2. Single channel recordings of a RyR2 channel fully activated by the combined action of cytosolic Ca^2+^ (10 µM) and caffeine (5 mM). Lumenal (*trans*) Ca^2+^ (50 mM) was the current carrier. All recordings were performed at holding voltage (V_m_) = 0 mV. Channel openings are observed as positives deflections of the current (*o* = full open; *b* = baseline). The frequency-current amplitude histograms obtained from 4-min single-channel recordings are shown next to each trace. Representative traces recorded before (top trace) and after addition of increasing concentrations of protamine (0.02, 0.2, 1, 2 and 20 µg/ml). Current levels of protamine-induced substates are indicated by dashed lines. Subsequent addition of 250 µg/ml heparin reversed the effect of protamine (bottom trace). 4 min-recordings were performed in all the above-mentioned conditions (n = 6 experiments).

As shown in Fig. 1, at high levels of Ca^2+^ and caffeine, increasing concentrations of protamine induced sub-conductance states (substates) of several current levels (indicated by dashed lines). With 0.02 µg/ml protamine, infrequent transitions to a substate of ∼50% of the full open conductance were observed. With 0.2 µg/ml protamine, the probability of this substate dramatically increased. Addition of protamine to reach a concentration of 1 µg/ml resulted in the formation of an additional substate of ∼20% the full open conductance. Under these conditions, the probability of full openings decreased to virtually zero and the channel fluctuated between the two substates. With 2 µg/ml protamine, the substate with the smaller conductance became predominant. It is possible that even smaller substates arise at high concentrations of protamine, but they cannot be unequivocally resolved from the baseline current noise. As previously reported for RyR1 [24], we observed full block of RyR2 in the presence of 20 µg/ml protamine. Addition of heparin (250 µg/ml), known to bind protamine with very high affinity [30], [31], [32], reversed the effect of protamine on RyR2. This reversibility suggests that the action of protamine was not related to the dissociation of any cofactor or modulatory subunit bound to the RyR2 channel.

**Figure 2:**
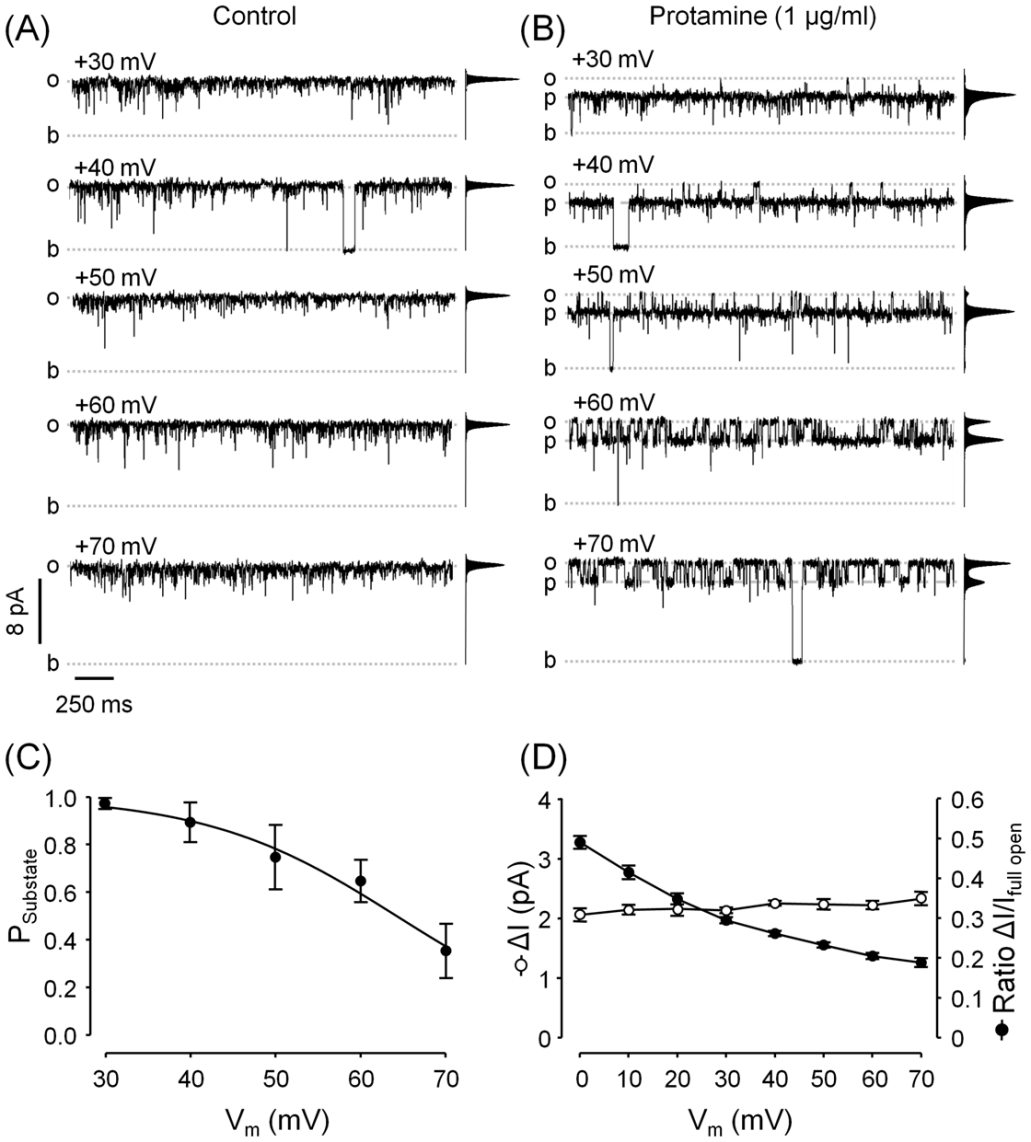
Effect of voltage changes on protamine-induced substates RyR2. Single-channel recordings of a RyR2 channel activated with Ca^2+^/caffeine (10 µM/5 mM, respectively). Openings are shown as upward deflections (*o* = full open; *b* = baseline). The frequency-current amplitude histograms obtained from 4-min single-channel recordings are shown next to each trace. Representative traces recorded at +30, +40, +50, +60 and +70 mV (*trans - cis*) are shown under control conditions (**A**) and after addition of 1 µg/ml protamine (**B**). *p* represents openings to the protamine-induced substate. (**C**) Fraction of time spent at the protamine-induced substate (P_Substate_) as a function of holding voltage (V_m_) ranging from +30 to +70 mV. Values are averages±SEM of P_Substate_ calculated from n = 5 recordings. Lines represent fits of the Woodhull equation to experimental data (see Results). (**D**) The drop in current amplitude from the full opening to the protamine-induced substate (ΔI) and the ratio: ΔI/I_full opening_ were calculated for 10 independent experiments and plotted as a function of holding voltage (open circles and filled circles, respectively). Averages ± SEM of these values are shown as a function of V_m_.

At 0 mV, Fig. 1 showed that 1 µg/ml protamine induced transitions between two subconductance levels. Figures 2A and 2B show that at positive voltages (SR lumen - cytosol), 1 µg/ml protamine induced the formation of substates of only one current level. The probability of this substate decreased with increasing positive holding voltages (Fig. 2C). This resulted in an increase of the probability of the full open state at higher voltages, as the channels were fully activated by the combined action of high cytosolic Ca^2+^ and caffeine prior to protamine addition.

As previously done [18], [33], we analyzed the voltage dependence of protamine block using a modified version of the equation derived by Woodhull [34] for block of a one-site, two-barrier channel model:
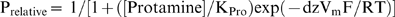
where F, R, and T have their usual meanings, P_relative_ is the ratio of open probabilities (presence of protamine/absence of protamine) measured at each voltage and K_Pro_ is the protamine concentration at which block is half-maximal at 0 mV. The “equivalent valence” of the blocker (dz) is influenced by z = 22 (average valence of protamine molecules) and d (fraction of the membrane potential acting at the site). This term includes binding site location within the membrane field, Ca^2+^ flow – protamine interactions and the possibility of multi-sites. Fitting of Woodhull equation to our data render a K_Pro_ = 50±23 ng/ml and d = 0.10±0.03. According to the Woodhull model, a d ∼0.1 would indicate that protamine interacts with a RyR2 region that “weakly senses the field”. This resembles previously reported observations for Imperatoxin A (IpTx_A_) [35] and neomycin [33]. Comparatively, peptide blockers [18] and even ryanodol, a neutral ryanoid [36], have sharper voltage-dependence.

**Figure 3:**
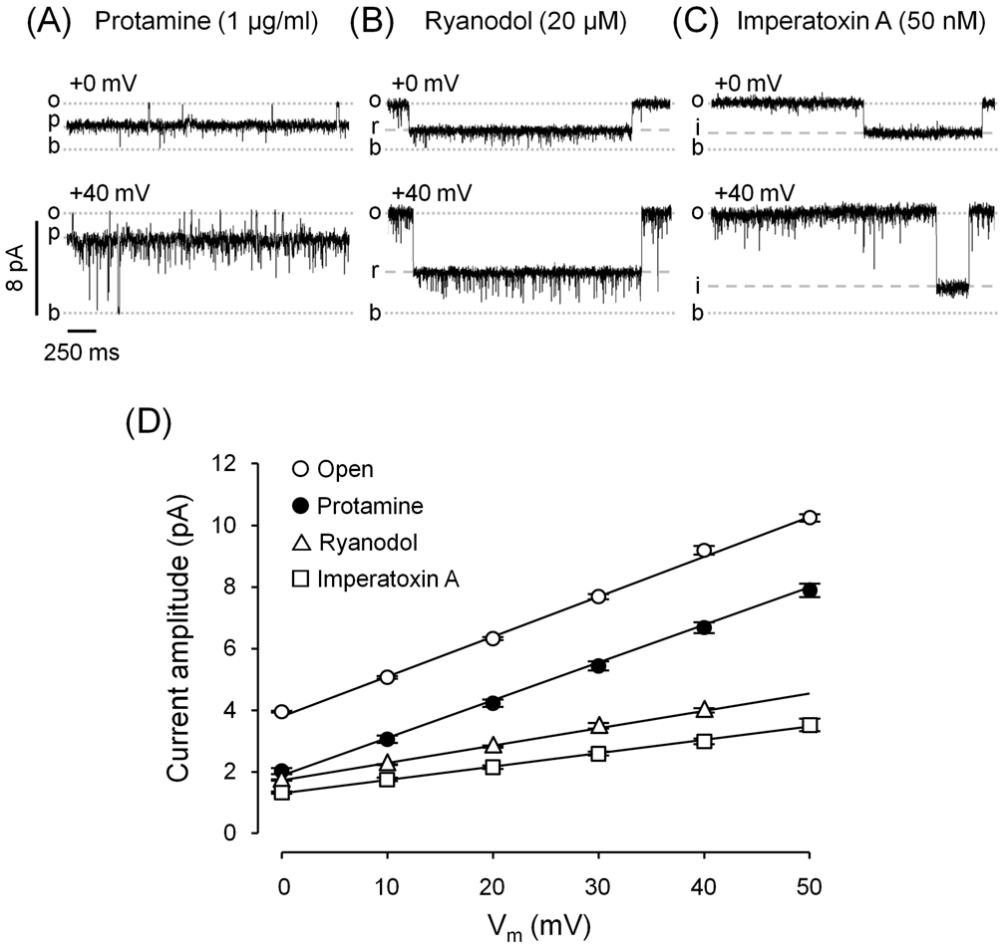
Effect of protamine compared to other conductance-modifiers. Single-channel recordings of fully active RyR2 channels (10 µM cytosolic Ca^2+^/5 mM caffeine) in the presence of 1 µg/ml protamine, 10 µM ryanodol or 50 nM imperatoxin A (**A**, **B** and **C**, respectively). Channel activity was recorded at V_m_ = 0 mV (top traces) and +40 mV (bottom traces). The current levels for the baseline, full open state and substates induced by protamine, ryanodol and IpTx_A_ are indicated (*b, o, p, r* and *i*, respectively). (**D**) Current amplitude as a function of voltage for the full open state (n = 28) and for substates induced by protamine (n = 10), ryanodol (n = 13) and IpTx_A_ (n = 5). Slope conductances were, in pS, 129±1, 123±3, 56±2 and 43±3, respectively.

As shown in Fig. 2B and 3A, the difference in current amplitude (full open – protamine substate) remains the same at all positive voltages. This is not the case for other known conductance-modifiers, where the substate amplitude changes proportionally with the amplitude of the full open channel. As an example, we show how a change in the holding voltage (from 0 to +40 mV) affects the amplitude of the substates induced by ryanodol and imperatoxin A (IpTx_A_) (Fig. 3B and 3C, respectively). Notice that at 0 mV ryanodol and IpTx_A_ induced the formation of substates which current levels of ∼45% and ∼33% the current through the full open channel. The same proportions were observed when the holding voltage was +40 mV. These results are reflected in the I-V curves were the slopes for the full open state, the ryanodol-induced substate and the IpTx_A_-induced substate are, respectively 129±1, 56±2 and 43±3 pS (Fig. 3D, open circles, triangles and squares). In contrast, the current levels of the protamine-induced substate at 0 mV and +40 mV were, respectively, ∼50% and ∼75% of the full open current. Since the drop in current induced by protamine is the same regardless of the holding voltage, the I-V curves for the open state and the protamine-induced substate display very similar slopes (129±1, 123±3 pS, respectively) (Fig. 3D, open circles vs. filled circles).

**Figure 4:**
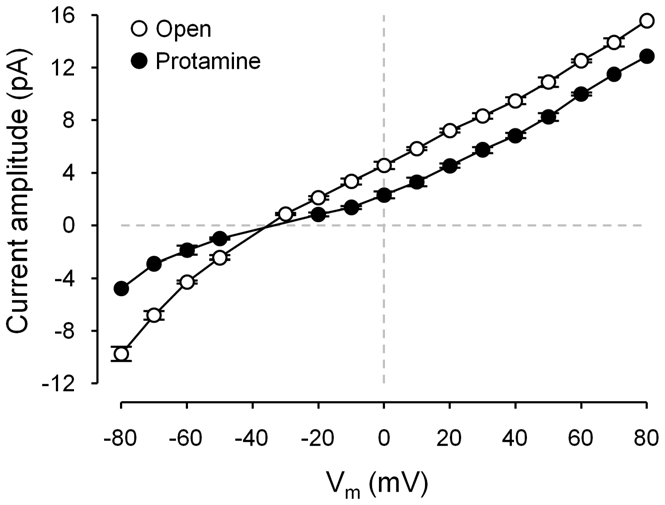
Current voltage (I-V) curves for the full open state and for the protamine-induced substate. Current amplitude as a function of voltage was plotted based on single-channel recordings of fully active RyR2 (10 µM cytosolic Ca^2+^/5 mM caffeine) performed in the presence of cytosolic Cs^+^ and 0.2 µg/ml protamine. Values are averages ± SEM of 5 experiments.

To determine the reversal potential for the full open state and the protamine-induced substate, experiments were conducted in the presence of Cs^+^ and Ca^2+^ in the *cis* and *trans* chambers, respectively. Figure 4 shows that the I-V curves for the full open state and for the protamine-induced substate meet at the same reversal potential. This indicates that the selectivity Ca^2+^/Cs^+^ during the full openings is the same as during protamine-modified events.

### Protamine Affects the Probability of Imperatoxin A and Ryanodol Substates

**Figure 5:**
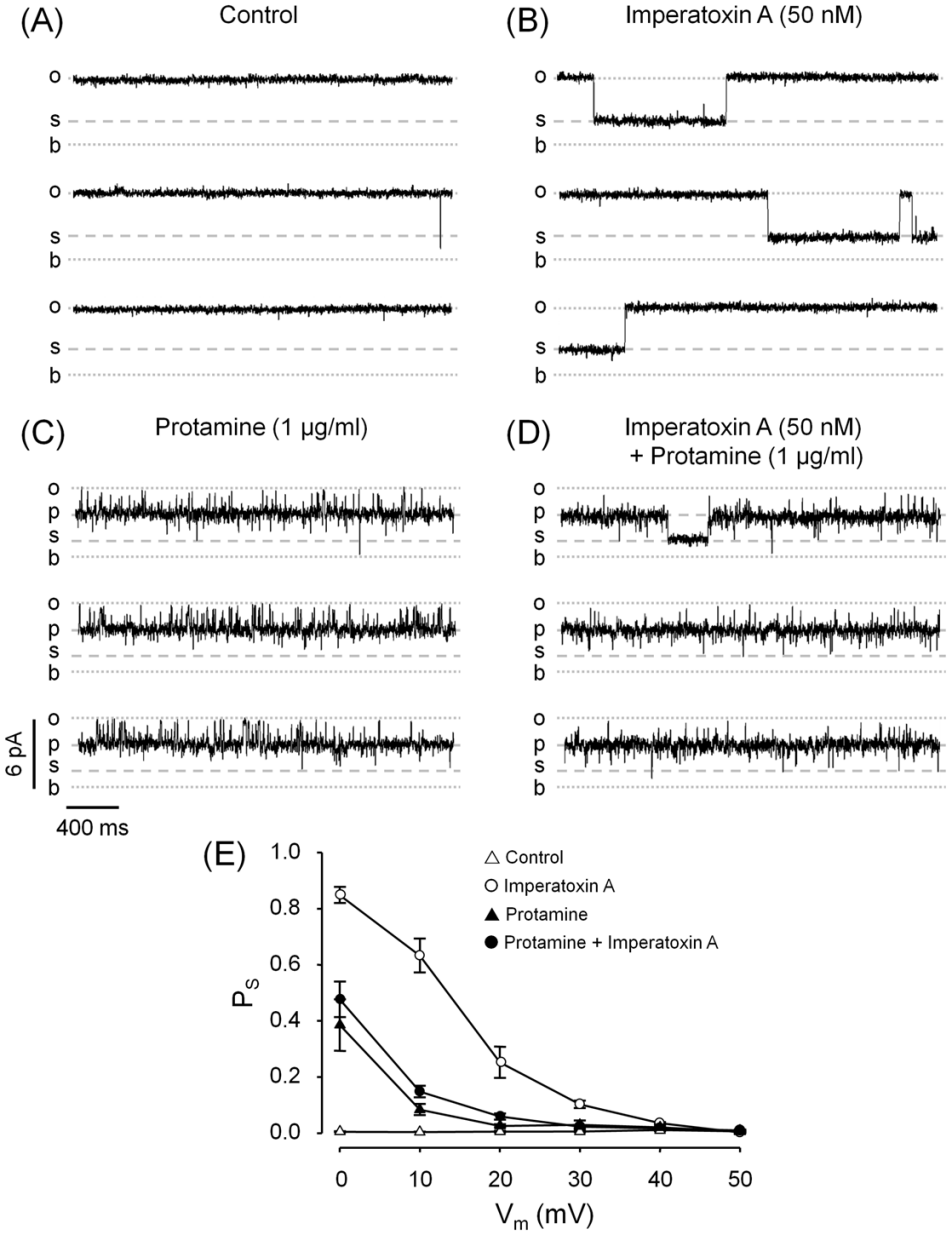
Effect of protamine in combination with imperatoxin A (IpTx_A_). RyR2 channels fully activated by the combined action of cytosolic Ca^2+^ (10 µM) and caffeine (5 mM) were used as control (**A**). Subsequently, 50 nM IpTx_A_ (**B**) or 1 µg/ml protamine (**C**) were added. To test the combined effect of 1 µg/ml protamine + 50 nM IpTx_A_ two sets of experiments were performed: one adding protamine first and then IpTx_A_ and the other, switching the order in which these agents were added. The results were the same in both sets of experiments. A representative recording is displayed in panel (**D**). All traces shown were performed at V_m_ = +20 mV. Current levels for the baseline (*b*), full open state (o), protamine-induced substate (*p*) are indicated. A small substate (*s*) is indicated. In panels (**A**) and (**B**) the amplitude of this substate is ∼30% of the full open state and in panels (**C**) and (**D**) it is ∼30% the amplitude of the protamine-induced substate. (**E**) Probability of substates at the *s* level (P_S_) as a function of holding voltage of RyR2 channels under control conditions (open triangles), exposed to 50 nM IpTx_A_ (open circles), 1 µg/ml protamine (filled triangles) and 50 nM IpTx_A_ +1 µg/ml protamine (filled circles). Values are means±SEM (* P<0.05; n = 10 experiments).

The peptide imperatoxin A (IpTx_A_) is an agent that has similarities to the II-III loop of DHPR [37], [38] and binds to a specific site at the open RyR to induce the formation of long-lasting, voltage dependent and reversible substates [35]. Here, we tested possible interactions between the sites responsible for the formation of protamine- and IpTx_A_-induced substates. We defined *“s”* as small substate that, in the absence of protamine, has ∼30% of the full opening current amplitude. In the presence of protamine, *s* is defined as the “sub-substate” and its amplitude is ∼30% of the protamine-induced highest conductance substate. As shown in Fig. 5A, no transitions to any substate were observed under control conditions (absence of IpTx_A_ and/or protamine). Figure 5B shows that at a holding voltage of +20 mV, IpTx_A_ alone induced frequent long-lived substates. The effect of 1 µg/ml protamine alone is shown in Fig. 5C: the channel spent most of the time in the protamine-induced substate with infrequent transitions to short lived sub-substates. The combined action of protamine and IpTx_A_ was tested in two sets of experiments, switching the order in which these agents were applied. The result was always the same regardless of which agent was added in the first place: the channel spent most of the time in the protamine-induced substate with infrequent transitions to sub-substates (Fig. 5D). In this case the sub-substates were kinetically heterogeneous: there were short-lived events, similar to those observed in absence of IpTx_A_ (Fig. 5C) and a few events of much longer duration that were only observed upon addition of IpTx_A_. These events could represent either IpTx_A_-induced substates in the protamine-modified channel or IpTx_A_ stabilization of the protamine-induced small-conductance substates. Still, the global probability of sub-substates (which includes both populations of events) was not significantly different in absence versus presence of IpTx_A_ (Fig. 5E, filled circles *versus* filled triangles). Thus, IpTx_A_ has little or no effect on RyR2 behavior when protamine is present.

**Figure 6:**
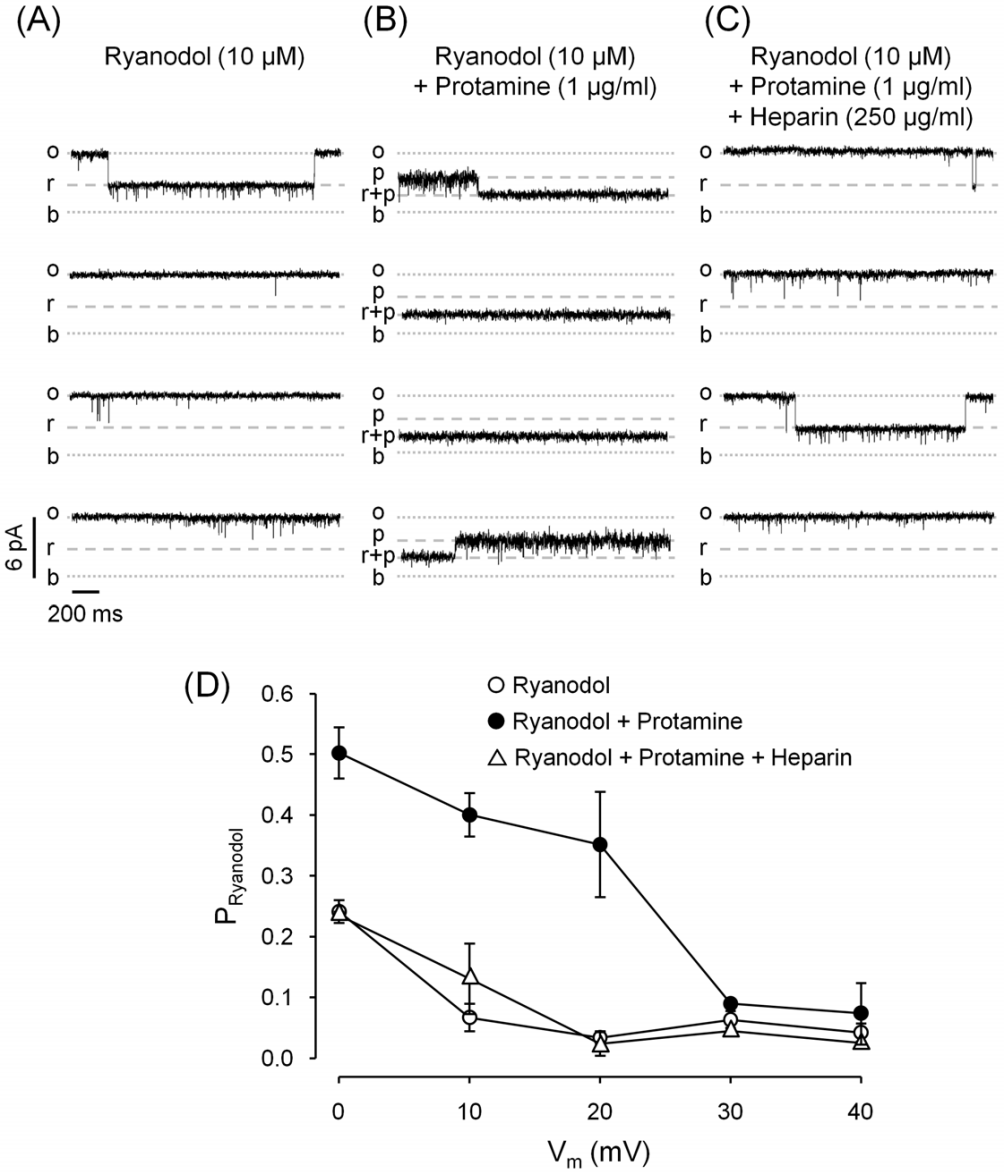
Effect of protamine in combination with ryanodol. Continuous trace of a RyR2 in the presence of 10 µM ryanodol before (**A**) and after subsequent addition of 1 µg/ml protamine (**B**) and 250 µg/ml heparin (**C**). Recordings were performed at V_m_ = +20 mV (*b = *baseline; *o* = full open state; *r* = ryanodol-induced substate; *p* = protamine-induced substate; *p+r* = ryanodol-induced substate on the protamine-modified RyR2). (**D**) Probability of ryanodol-induced substates (P_Ryanodol_) as a function of holding voltage of RyR2 channels exposed to 10 µM ryanodol (open circles); 10 µM ryanodol+1 µg/ml protamine (filled circles) and 10 µM ryanodol+1 µg/ml protamine+250 µg/ml heparin (open triangles). Under these three conditions, the dwell times of the ryanodol-induced events were (in seconds): 3.7±0.7; 11.3±2.5 and 3.4±0.5 respectively. Values are means±SEM (* P<0.05; n = 7 experiments).

Ryanodol binds to open RyRs at a specific “ryanoid site” inducing the formation of reversible substates of ∼45% of the full open channel conductance. Previous studies indicated that the ryanoid site senses the electric field [36], [39]. Here, we tested the possibility that protamine induces substates by reversibly interacting with the “ryanoid site”. If this is the case, then we should not observe protamine-induced substates in ryanodol-modified channels (the “ryanoid site” would be unavailable to protamine when it is occupied by ryanodol). As shown in Fig. 6A, in the presence of 10 µM ryanodol, RyR2 reversibly fluctuated between the full opening and the ryanodol-modified state. After addition of protamine (Fig. 6B) the channel displayed virtually no full openings and it alternated between the protamine-induced substate and a long lasting (11±3 seconds at Vm = 20 mV) smaller substate which was never observed in the absence of ryanodol. Thus, protamine and ryanodol are not likely to act at the same binding site given that their effects do not exclude each other. However, in the presence of protamine, the ryanodol effects appear to be potentiated as events are longer and more frequent than those observed in the absence of protamine (Fig. 6B, legend). Therefore, addition of protamine increased the probability of ryanodol-induced substates (P_Ryanodol_) (Fig. 6D). As expected, subsequent addition of heparin reversed this effect, the channel turned back to fluctuate between the full open state and the ryanodol substate (Fig. 6C), and P_Ryanodol_ decreased to control values (Fig. 6D).

### Protamine Increases the Activity of RyR2 at Low Cytosolic Ca^2+^

**Figure 7:**
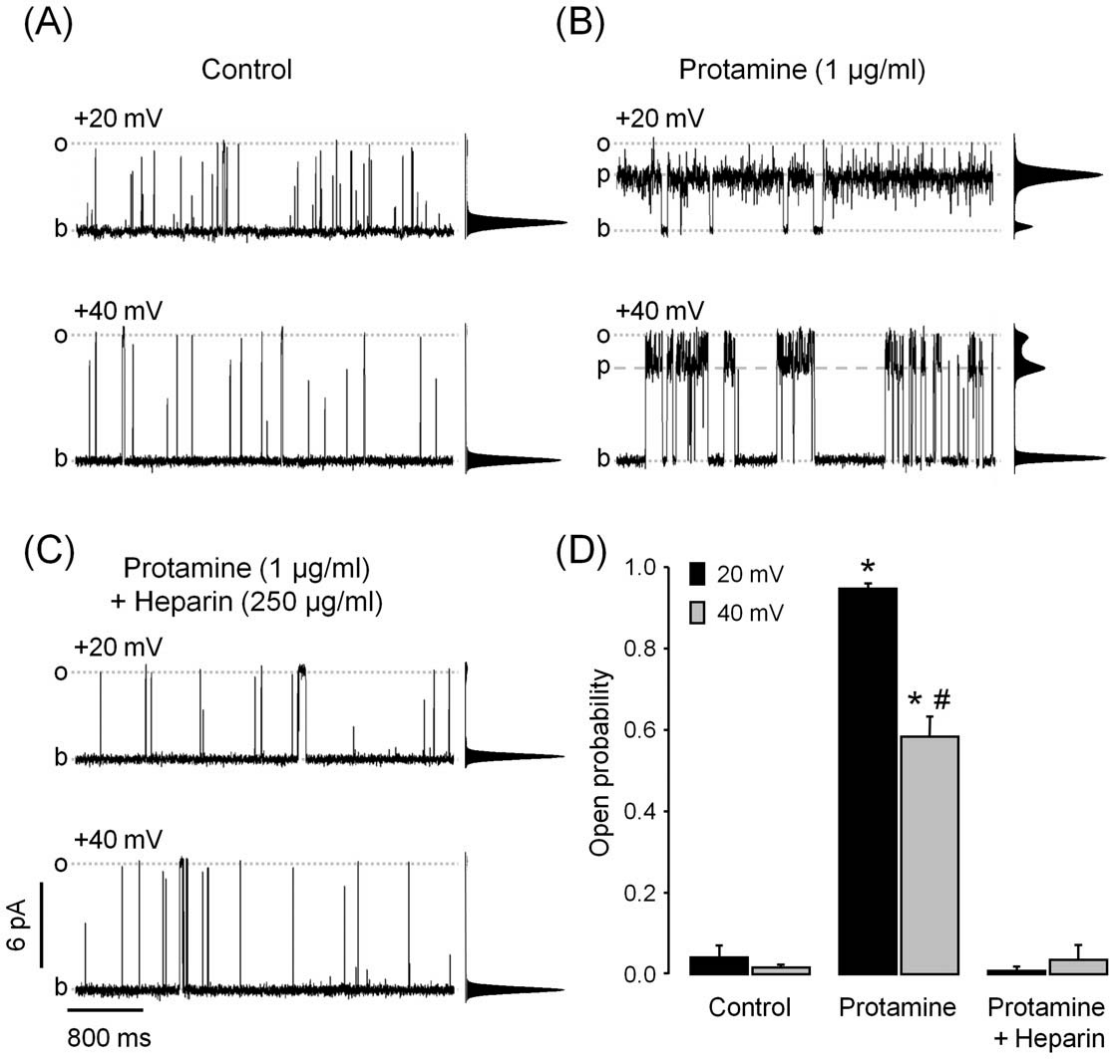
Effect of protamine at low cytosolic Ca^2+^. Single channel recordings of partially active RyR2 in the presence of 100 nM cytosolic Ca^2+^ before (**A**) and after subsequent addition of 1 µg/ml protamine (**B**) and 250 µg/ml heparin (**C**). The frequency-current amplitude histograms obtained from 4-min single-channel recordings are shown next to each trace. Channel activity was recorded at V_m_ = +20 mV (top traces) and +40 mV (bottom traces). (**D**) Averages of open probabilities calculated for single RyR2 at V_m_ = +20 mV (black columns) and +40 mV (shaded columns). Here, the open probability includes the probability of the full opening plus the probability of protamine-induced substates. Values are shown as means±SEM (* P<0.05 versus control; # P<0.05 versus protamine at +20 mV; n = 5 experiments).

We tested the effect of protamine on RyR2 with low P_o_ in the presence of 100 nM cytosolic Ca^2+^ as this condition better mimics the channel environment in a resting cell. In the control, at V_m_ = 20 mV, a few short and infrequent openings were observed (Fig. 7A) and the open probability (P_o_) was 0.042±0.029 (Fig. 7D). Upon addition of 1 µg/ml protamine, most of the openings were to the protamine-induced substate (Fig. 7B) reaching a P_o_ = 0.946±0.013 (Fig. 7D). As expected, upon addition of heparin, the protamine-induced substate was no longer observed (Fig. 7C) and channel activity returned to the levels observed under control conditions, with a P_o_ = 0.009±0.006 (Fig. 7D). Protamine activating effect on RyR2 was voltage-dependent and P_o_ only reached 0.583±0.050 at V_m_ = 40 mV (Fig. 7D). The activating effect of protamine was not affected by further decreasing cytosolic Ca^2+^ levels to 25 nM (P_o_ = 0.950±0.012 and 0.901±0.079, before and after decreasing Ca^2+^ levels, respectively; V_m_ = 20 mV; n = 5). Addition of 5 mM cytosolic Mg^2+^ decreased the amplitude of the full open state as well as the substate by ∼15% at 20 mV, which is expected from the decrease in driving force for divalent flux [29]. However, Mg^2+^ did not affect protamine induced activation (P_o_ = 0.961±0.006 and 0.951±0.018, before and after addition of Mg^2+^, respectively; V_m_ = 20 mV; n = 4). Notice that in the absence of protamine, at low cytosolic Ca^2+^ and high Mg^2+^, RyR2 channels had P_o_∼0, even when exposed to 10 mM caffeine [40], [41]. The activating effect of protamine did not require lumen-to-cytosol Ca^2+^ flux as it was also observed after replacing lumenal Ca^2+^ with Ba^2+^ (not shown), a divalent cation that does not activate RyR2 [42].

**Figure 8:**
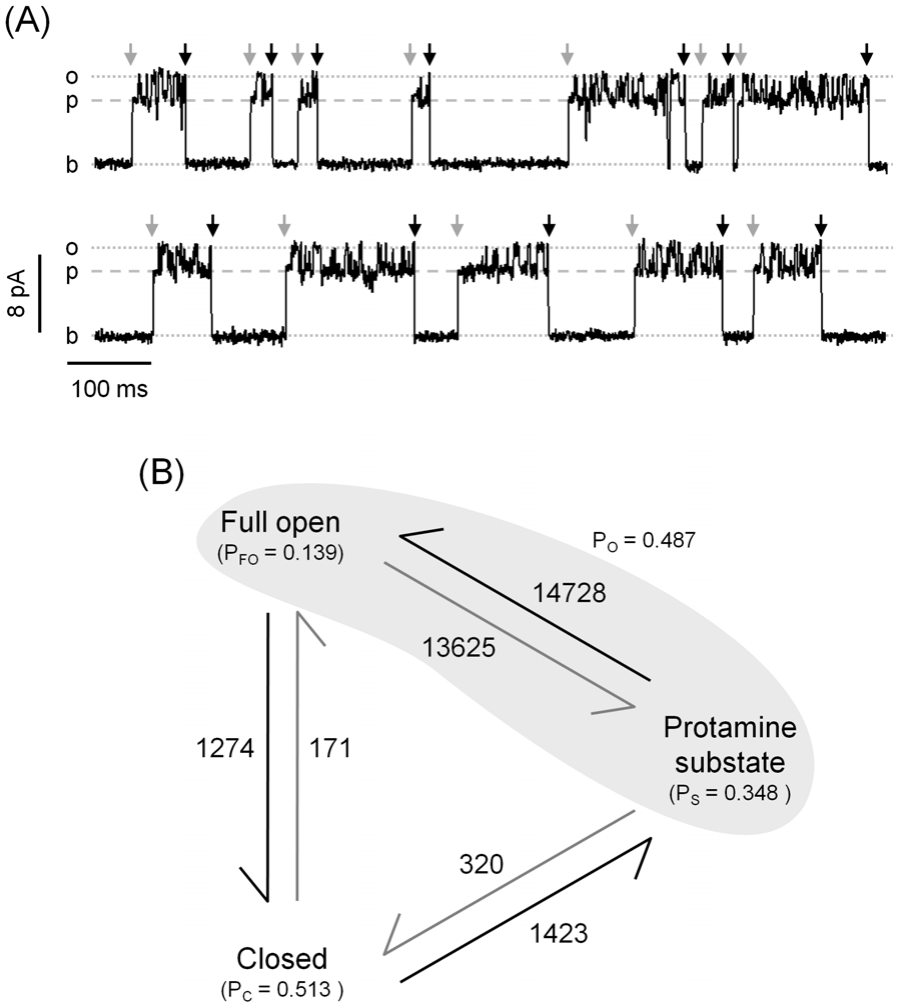
Sequence of events for protamine-induced activation of RyR2. (**A**) Continuous trace of a RyR2 in the presence of 25 nM cytosolic Ca^2+^ plus 1 µg/ml protamine recorded at V_m_ = +40 mV (*b* = baseline; *o* = full opening; *p* = protamine-induced substate). The beginning and end of each opening are indicated by grey and black arrows, respectively. (**B**) Diagram of states of RyR2 exposed to 1 µg/ml protamine with 25 nM cytosolic Ca^2+^ (4 min recording at V_m_ = +40 mV). The following states were considered: full open, protamine substate, closed. State probabilities (P) are given in parenthesis. Arrows represent observed unidirectional transitions from one state to another. Adjacent to each arrow is the respective absolute number of transitions.

Figure 8A shows a continuous recording of a RyR2 in the presence of protamine at low cytosolic Ca^2+^. Notice that when the RyR2 activates, it preferentially switches from the closed state to the protamine-induced substate rather than to the full open state (Fig. 8A, grey arrows). In contrast, when the channel closes, it is more likely to do it from the full open state than from the protamine-induced substate (Fig. 8A, black arrows). Estimations (from 4 minutes of recordings at +40 mV) of the probability of full openings, closures and protamine-induced substates are shown in Fig. 8B. Analysis of these results suggests that there is a preferential sequence of events during protamine-induced RyR2 activation. First, protamine binds to the closed channel and induces activation. Transitions from closed to the substate (1423) are 8.3 times more probable than transitions from closed to full openings (171). Once activated, the channel fluctuates between the substate and the full open state, probably as a result of rapid binding-unbinding of protamine to its site. Relative rate of transition (number of transitions A→B/probability A) for Full open→Closed (9165) is nearly ten-fold that for Protamine substate→Closed (919). If we assume that protamine is unbound during full openings, then the RyR2 would be no longer under the activating influence of protamine and it would be more likely to close.

## Discussion

Protamine did not simply block RyR2 in an all-or-none fashion. Rather, it affected the RyR2 channel conduction pathway by producing substates and full block in a voltage-dependent and dose-dependent manner. At low concentrations, protamine induced a drop in the current amplitude to a large conductance substate. The ratio between this amplitude drop and the amplitude of the full opening decreases as the positive holding voltage (V_m_) increases. This is reminiscent of the rectification of some K^+^ channels observed in presence of polyamines. Furthermore, protamine activated channels at low resting cytosolic Ca^2+^ levels (25–100 nM) even in the absence of lumenal Ca^2+^ or in the presence of high cytosolic Mg^2+^ levels, suggesting that protamine-induced RyR2 activation mainly results from electrostatic interactions and does not seem to require cytosolic or luminal Ca^2+^ binding to the respective activating sites.

### Comparison with Previous Studies

In this work, protamine added to the cytosolic surface of RyR2 had a complex action as it affected RyR2 channel conduction (multiple substates/block) and gating (activation). Our observation of full block of RyR2 induced by high levels of protamine (20 µg/ml; V_m_ = 0 mV) are consistent with previous findings in single RyR1 channels [24]. However, lower protamine concentrations that would have allowed to observe protamine-induced RyR1 activation have not been tested in this previous report [24]. Moreover, the substates and full block of fully activated RyR2 observed here at V_m_ = 0 mV would lead to a decrease in current flow through the channels, which is in agreement with previous reports were protamine was found to inhibit caffeine-induced RyR1-mediated Ca^2+^ release from skeletal muscle SR microsomes [19] (notice that the expected SR lumen voltage in microsomes is ∼0 mV). Although this previous study did not test the effect of protamine in conditions homologous to those used here to detect activation, it provided evidence that RyRs can be activated by another polyarginine (Histone IIS) and by a synthetic polylysine [19]. These agents were found to activate Ca^2+^ release from resting RyR1 and to induce block of drug-activated channels [19], [20]. Similarly, polylysine has been reported to activate RyR2-mediated Ca^2+^ release in cardiac microsomes regardless of the presence/absence of Mg^2+^ [43].

The literature suggests that the activation of RyR by polycations is relatively restricted to peptides like polylysines and polyarginines [19], [20]. Protamine/histone/polylysine-induced activation of SR Ca^2+^ release would result from Ca^2+^-independent and Mg^2+^-insensitive transitions from closed to the high subconductance state in resting RyRs. Protamine and polylysine have been reported to produce diverse effects that do not include activation on other ionic channels [44], [45], [46]. Thus, protamine- and polylysine-induced activation seems to be specific for RyRs. Inhibition of drug-stimulated RyR-mediated SR Ca^2+^ release would result from voltage-dependent transitions from the full open state to multiple subconductance and block-like states. A large variety of polycationic molecules were found to produce these effects on RyRs [3], [47], [48], [49] as well as on other channels [50], [51], [52], [53].

### Effect of Protamine on RyR2 Channels Conductance

Protamine is essentially a poly-arginine molecule with a charge of ∼+22 [25]. Thus, it is reasonable to expect voltage-dependent effects on the RyR channels as they contain rings of negatively charged amino acids in the cytosolic vestibular regions that sense the electrical field [16], [17]. Indeed, at V_m_≤0 mV protamine added to the RyR cytosolic surface produced several subconductance states as well as full block. However, at V_m_ ranging from +10 to +80 mV, block events and smaller substates disappeared, suggesting sharp voltage-dependence, while the substate with the highest level of conductance remained with a significant probability. This difference in the rate of decline of the probability for the different substates could indicate that multiple protamine molecules, despite their large charge and size, would bind to multiple sites in the RyR vestibule that would have differential sensitivity to the electric field. This would be in agreement with the apparent multiple binding components that have been suggested for polyamine-induced rectification of channels [50], [51], [52], [53].

The nature and functional implication of substates in RyRs is not known (discussed in [18]). Protamine substates are different from those induced by known RyR conductance modifiers such as ryanoids and Imperatoxin A (IpTx_A_) [3], [18], [35], which change channel conductance by inducing a drop in the current amplitude that is proportional to the amplitude of the full opening (i.e., these agents produce a “rigid” conformational change in the RyR's conduction pathway). The nearly constant drop in current amplitude observed at positive voltages for the protamine-induced transition from full opening to substate is unusual and it implies that the proportion by which the conductance is reduced (relative to the size of the full open channel) is less as the holding voltage increases. As the driving force of Ca^2+^ fluxes increases, it could better counteract an electrostatic screening phenomenom produced by bound protamine and/or change the orientation of protamine putative coiled-coil structure in a rigid vestibule [54]. It is also possible that protamine binds to a flexible loop in the RyR that modulates the ability of protamine to act as a plug within the vestibule in a voltage-dependent manner.

The cationic peptide IpTx_A_, induces long-lived substates by binding to sites putatively located at RyR regions that sense the electrical field [35]. More recently, a 3D mapping study has located the IpTx_A_ binding to a RyR region between the clamp domain and the central handle domain [55]. Here, protamine greatly decreased the probability of IpTx_A_-induced substates suggesting that protamine may bind to the same RyR region as IpTx_A_.

Protamine was effective at inducing substates in ryanodol-modified channels, suggesting that the binding sites for protamine and for ryanoids do not overlap. The ryanodol-protamine interactions seem to be cooperative since protamine increased the probability of ryanodol-induced substates. This result resembles previously reported findings, where IpTx_A_, a segment of the DHPR II-III loop (Peptide A) and other positively charged “ball peptides” affected the action of ryanodol in a cooperative fashion [18]. As ryanoids and IpTx_A_ were shown to bind to different regions in the RyR molecule [55], [56], our finding that protamine affects the action of both agents would suggest multiple sites of action or allosteric effects. Increase in ryanodine binding induced by polylysine has also been reported for skeletal and cardiac RyRs [20], [21], [23].

In summary, protamine affects RyR2 channel conduction (substates and block) and the binding affinity of agents that interact with vestibular regions of RyR2. This could be the result of allosterically-induced flexible conformational changes, although steric interference cannot be ruled out. This effect of protamine on conduction does not appear to be highly specific as similar effects were found for other polycationic molecules [3], [18], [47], [48], [49].

### Protamine Activates RyR2

Protamine (1 µg/ml) activated RyR2 at cytosolic Ca^2+^ levels below those found in resting cells (when the RyR2 are normally closed). Regarding the steps involved in protamine-induced activation, our results show that there is a preferential sequence of events. The channels first open to reach the protamine substate level and then they fluctuate between the full open sate and the substate. We also observed that the probability to close is higher when the channel is fully open than during substates. Like protamine, IpTx_A_- and ryanoids-induced substates rarely transition to the closed state [34], [37]. Unlike protamine, IpTx_A_ and ryanoids do not activate RyR2 under resting Ca^2+^ conditions [1], [3], [18], [33], [34], [37].

The efficacy of protamine to activate RyR2 was not affected by addition of up to 5 mM Mg^2+^, known to compete with Ca^2+^ at the RyR2 cytosolic binding activating sites [1], [3], [42]. Moreover, protamine remained effective after we replaced lumenal Ca^2+^ with Ba^2+^, which would prevent “feed through” activation, as Ba^2+^ can permeate through the channel but does not activate it [42]. The results support the idea that protamine-induced activation of RyR2 is mostly mediated by interactions that are Ca^2+^- independent within levels found in the cytosol and SR lumen of myocytes.

Although RyRs are highly sensitive to the activating action of protamine and polylysine, they are not gated open by a variety of other cationic molecules, including peptides, polyamines and macrolide antibiotics, which only produce substates and/or block [18], [47], [48], [49]. The reason for this differential sensitivity is still unclear but it is possible that the high efficacy of cationic polypeptides is related to the large magnitude of their charge density (e.g., ∼+22 for protamine), which would allow them to strongly interact with a negatively charged gating domain. Previous studies have found that Ca^2+^ dependence of RyR gating is insensitive to carbodiimide titration of negative surface charges located either on the luminal or the cytosolic channel surface [57]. Likewise, [^3^H]ryanodine binding remained Ca^2+^ dependent even in presence of very high salt levels (1 M NaCl or KCl) which should greatly affect electrostatic interactions [58]. Thus, it is possible that the action of protamine may be more than just a surface-charge effect. Accordingly, previous reports with histones and polylysines of different molecular weights and charges did not clearly indicate whether the most charged molecules are better RyR activating agents [19]. In principle, poly L-arginines would be better activating agents than poly L-lysines [59]. Indeed, we reach near maximal activation of RyR2 with ∼250 nM protamine, which is about one tenth of the concentration of a larger molecular weight polysysine required to elicit the same effect [19], [52]. It is possible that the arginines present in protamine are more suitable to form strong π-cation interactions with aromatic aminoacids present at the RyR gating domain, resulting in a higher binding affinity [60].

### Is There a Role for Modulation of RyRs through Electrostatic Interactions

Protamine is clinically utilized to reverse heparin overdose and has been shown to display significant cardiotoxic effects, including decreased cardiac output and electrocardiographic disturbances [31]. Studies performed using isolated myocytes revealed multiple effects of protamine, including changes in cardiomyocyte contraction (depressed contraction at 3 Hz but enhanced at 0.5 Hz or at lower rates), partial membrane depolarization, rise in resting tension and appearance of rested state rapid cooling contractures [61]. The effects of protamine have been associated to calcium overload and impairment of sarcoplasmic reticulum functions [62]. Our results open the possibility that direct protamine binding to RyR2 could mediate, at least in part, the observed abnormalities in SR Ca^2+^ homeostasis.

It is plausible that the strong effect of protamine on channel conduction and gating results from its interaction with the rings of charges in the vestibular region of RyRs [63]. However, the physiological role for the existence of a RyR channel domain that confers susceptibility to modulation by agents like protamine is still unknown. A physiological implication of our findings is the possibility that protamine mimics the action of positively charged peptide motifs, present in the vicinity of the RyR2 channels in cells which might modulate channel activity (activation and deactivation). In favor of this possibility, previous observations suggested that RyRs hypersensitivity to polylysine is associated with cardiac diseases [22] or with modifications of skeletal RyR1 interdomains that trigger malignant hyperthermia [23].

Studies of multichannel behavior indicated that physical RyR-RyR interactions between domains of neighboring channels could shape their coupled gating [8], [11], [12], [14]. It is possible that protamine-sensitive domains are involved in this process. RyR also interact with DHPRs. Indeed, during the action potential, physical DHPR-RyR1 interactions activate RyR1-mediated Ca^2+^ release in skeletal fibers [2].

The very limited effects of IpTx_A_ and DHPR peptides on sparks suggest they have restricted access to the RyR2 [64]. An interesting observation is that protamine also has very limited effect on RyR1 in skeletal fibers, suggesting that the protamine-sensitive domains of RyR1 are not accessible in the cellular environment [65]. An interpretation is that there might be an endogenous polycationic peptide, which could move when propelled by a change in the electric field at the T-tubule during excitation to induce activation of RyR1. The same moiety could subsequently block the RyR1 when the T-tubule voltage returns to resting values. In the heart, Ca^2+^ entry is known as an absolute requirement for cardiac EC coupling [5] and the presence of a voltage-dependent component in the activation is still an open question [66]. Still, the observation of Ca^2+^-entry-independent negative modulation of local events of Ca^2+^ sparks by specific DHPR agonist and blockers suggests the existence of physical RyR2-DHPR interactions of a still unknown nature that regulate RyR2 function [5], [15]. The protamine-binding domain in RyR2 might be involved in this modulation.

In summary, our studies indicate that protamine, can induce Ca^2+^-independent RyR2 activation and deactivation. The possibility of similar moieties shaping RyR-RyR interactions for functional coupling or DHPR-RyR interactions for triggering and/or terminating SR Ca^2+^ release opens the field for further investigation.
